# Abolition of Peroxiredoxin-5 Mitochondrial Targeting during Canid Evolution

**DOI:** 10.1371/journal.pone.0072844

**Published:** 2013-09-02

**Authors:** Valérie Van der Eecken, André Clippe, Sophie Dekoninck, Julie Goemaere, Geoffroy Walbrecq, Paul P. Van Veldhoven, Bernard Knoops

**Affiliations:** 1 Institut des Sciences de la Vie, Université catholique de Louvain, Louvain-la-Neuve, Belgium; 2 Department of Cellular and Molecular Medicine, LIPIT, Katholieke Universiteit Leuven, Leuven, Belgium; Instituto de Biociencias - Universidade de São Paulo, Brazil

## Abstract

In human, the subcellular targeting of peroxiredoxin-5 (PRDX5), a thioredoxin peroxidase, is dependent on the use of multiple alternative transcription start sites and two alternative in-frame translation initiation sites, which determine whether or not the region encoding a mitochondrial targeting sequence (MTS) is translated. In the present study, the abolition of PRDX5 mitochondrial targeting in dog is highlighted and the molecular mechanism underlying the loss of mitochondrial PRDX5 during evolution is examined. Here, we show that the absence of mitochondrial PRDX5 is generalized among the extant canids and that the first events leading to PRDX5 MTS abolition in canids involve a mutation in the more 5′ translation initiation codon as well as the appearance of a STOP codon. Furthermore, we found that PRDX5 MTS functionality is maintained in giant panda and northern elephant seal, which are phylogenetically closely related to canids. Also, the functional consequences of the restoration of mitochondrial PRDX5 in dog Madin-Darby canine kidney (MDCK) cells were investigated. The restoration of PRDX5 mitochondrial targeting in MDCK cells, instead of protecting, provokes deleterious effects following peroxide exposure independently of its peroxidase activity, indicating that mitochondrial PRDX5 gains cytotoxic properties under acute oxidative stress in MDCK cells. Altogether our results show that, although mitochondrial PRDX5 cytoprotective function against oxidative stress has been clearly demonstrated in human and rodents, PRDX5 targeting to mitochondria has been evolutionary lost in canids. Moreover, restoration of mitochondrial PRDX5 in dog MDCK cells, instead of conferring protection against peroxide exposure, makes them more vulnerable.

## Introduction

Due to the presence of the electron transport chain, mitochondria are particularly exposed to oxidative attacks caused by reactive oxygen species (ROS) and reactive nitrogen species (RNS). To scavenge ROS/RNS, the mitochondrial matrix is equipped with antioxidant enzymes. Among them, manganese superoxide dismutase (MnSOD or SOD2) is responsible for the dismutation of the superoxide radical (O_2_ **^•^** ^–^) to hydrogen peroxide (H_2_O_2_). H_2_O_2_ is subsequently reduced to water by mitochondrial peroxidases, i.e. glutathione peroxidase 1 (GPX1), glutathione peroxidase 4 (GPX4), peroxiredoxin-3 (PRDX3) and peroxiredoxin-5 (PRDX5) [1].

PRDX5 is a thiol-specific peroxidase able to reduce H_2_O_2_, alkyl hydroperoxides and peroxynitrite [2]–[5]. The importance of PRDX5 in antioxidant defence was demonstrated in several studies, reviewed in [6]. The subcellular distribution of human PRDX5 is not restricted to mitochondria, as it is also localized in the cytosol, the peroxisomes and, in some situations, in the nucleus [2], [3], [7]–[9]. PRDX5 uses cytosolic and mitochondrial thioredoxins (TXNs or Trxs) as physiological electron donors. In turn, TXNs are reduced by mitochondrial or cytosolic thioredoxin reductases (TXNRDs or TrxRs), finally relying on NADPH as electron donor [3]–[5], [10].

**Figure 1:**
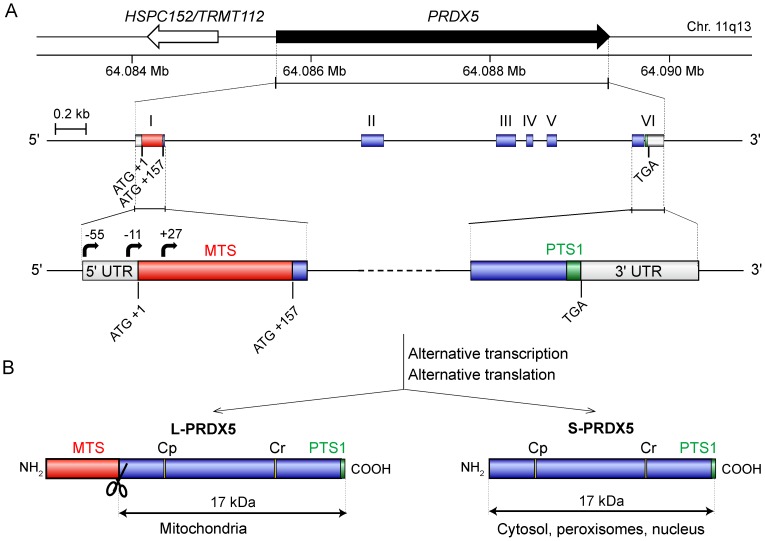
Human *PRDX5* gene organisation and protein forms. (A) Human PRDX5 gene is located on chromosome 11q13 and is flanked by the *HSPC152/TRMT112* gene, positioned in opposite direction. Human *PRDX5* contains six exons (boxes) and five introns. 5′ and 3′ UTRs are indicated by open boxes. In human liver, three alternative transcription initiation sites were identified (black arrows) [11]. Exon I contains two in-frame ATG initiation codons. The regions encoding the mitochondrial targeting sequence (MTS) and peroxisomal targeting signal (PTS1) are in red and green, respectively. (B) The presence of alternative transcription and translation start sites in human *PRDX5* gene results in the production of a long PRDX5 form (L-PRDX5) and a short PRDX5 form (S-PRDX5). L-PRDX5 contains a cleavable (scissors) N-terminal MTS (red) and is therefore imported into mitochondria. S-PRDX5 contains a weak C-terminal PTS1 (green) and is targeted to the cytosol and the peroxisomes. Peroxidatic (Cp) and resolving (Cr) Cys implicated in catalysis are indicated. Adapted from [6], [11].

**Figure 2:**
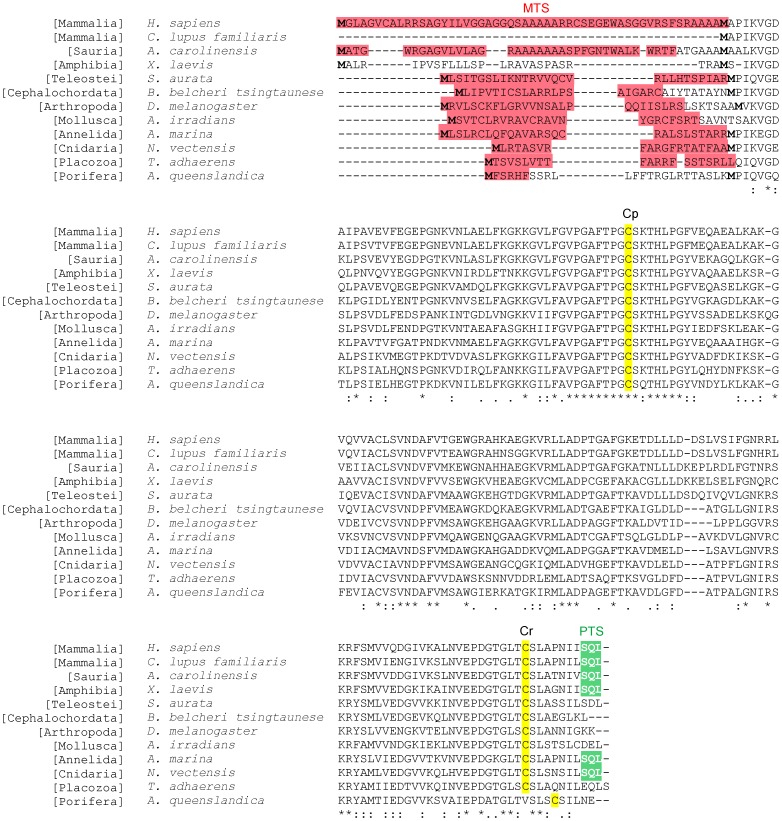
ClustalX 2.0.7 amino acid sequence alignment of human PRDX5 and animal orthologs. Asterisks (*) indicate identities, dots (.) and double dots (:) indicate conservative and highly conservative substitutions, respectively. Methionines encoded by the two alternative translation initiation sites are indicated in bold type. Peroxidatic (Cp) and resolving (Cr) Cys implicated in catalysis are highlighted in yellow. The peroxisomal targeting signal (PTS1) is highlighted in green. Mitochondrial targeting sequence (MTS) predictions were performed with TargetP [22], [43] and Mitoprot [21]. MTS predicted by TargetP are highlighted in red. MitoProt and TargetP subcellular localization predictions for PRDX5 were similar, except for *X. laevis* PRDX5, which was predicted to be imported into mitochondria with high probabilities when using Mitoprot, while the protein was predicted to be secreted with TargetP. Taxonomic groups are indicated between brackets. GenBank accession numbers are NP_036226.1 (human, *Homo sapiens*); XP_533241.1 (domestic dog, *Canis lupus familiaris*); XP_003230134.1 (green anole, *Anolis carolinensis*); NP_001085580.1 (African clawed frog, *Xenopus laevis*); ADI78068.1 (gilthead seabream, *Sparus aurata*); AAM18076.1 (Japanese lancelet, *Branchiostoma belcheri tsingtaunese*); NP_650679.3 (fruitfly, *Drosophila melanogaster*); ADQ57291.1 (bay scallop, *Argopecten irradians*); AAY96293.1 (lugworm, *Arenicola marina*); XP_001629337.1 (Starlet sea anemone, *Nematostella vectensis*); XP_002108078.1 (*Trichoplax adhaerens*); XP_003384607.1 (*Amphimedon queenslandica*).

In human, the complex subcellular distribution of PRDX5 is a consequence of the existence of two protein isoforms. The latter are encoded by a single gene containing alternative transcription start sites and two alternative in-frame translation initiation sites (Figure 1). Translation at the more 3′ start codon produces a short PRDX5 form (S-PRDX5) which is found in the cytosol, the peroxisomes and the nucleus [11]. Peroxisomal targeting is linked to the presence of a weak C–terminal peroxisomal targeting signal (PTS1) [2], [7]. Translation at the more 5′ start codon produces a long PRDX5 form (L-PRDX5) containing an additional N-terminal mitochondrial targeting sequence (MTS) or presequence [2], [11]. This MTS is cleaved after mitochondrial import, releasing the mature mitochondrial PRDX5 which is identical to S-PRDX5. Moreover, mitochondrial targeting of PRDX5 seems to be a conserved feature in animal kingdom. So far, the mitochondrial localization of the protein was experimentally verified in human, rat, mouse, hamster, bovine and fruitfly [2], [12]–[16]. Furthermore, presequences were also identified in orthologous PRDX5 of invertebrate species [16]–[19] (see also Figure 2). Some of these invertebrate species belong to basal metazoan phyla, i.e. porifera and placozoa. However, we reported recently that mitochondrial PRDX5 is absent in some mammalian species, such as in pig [19]. Moreover, according to sequence analysis, we also predicted that mitochondrial PRDX5 would be absent in dog (Figure 2) [11]. While the present work was in progress, the absence of dog L-PRDX5 was also evidenced by [20].

Here, we confirm that dog PRDX5 is not addressed to mitochondria and we examined the molecular basis responsible for the abolition of mitochondrial targeting. Furthermore, we show that the loss of mitochondrial PRDX5 is generalized throughout the Canidae family. Finally, the functional consequences of the restoration of mitochondrial PRDX5 in dog MDCK cells were also investigated.

## Results

### Mitochondrial PRDX5 is Absent in Domestic Dog

**Figure 3:**
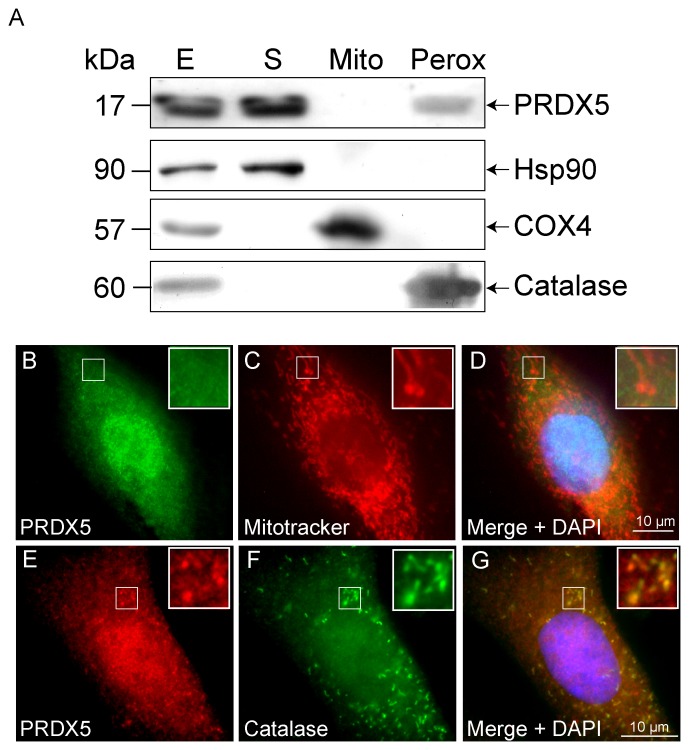
Dog PRDX5 subcellular distribution. (A) Intracellular localization of dog PRDX5 was assessed by liver subcellular fractionation. Post-nuclear (E), cytosolic (S), mitochondrial (Mito) and peroxisomal (Perox) fractions were analyzed for marker enzyme and PRDX5 content by Western blotting, using anti-Hsp90 (cytosolic), anti-COX4 (mitochondrial), anti-catalase (peroxisomal) and anti-PRDX5 antibodies. Fifteen µg of proteins from each fraction were loaded. PRDX5 subcellular localization was also assessed by immunofluorescence detection of endogenous PRDX5 in MDCK cells (B, E) with co-localization with MitoTracker Red (C) or with peroxisomal catalase (F). Nuclei were counterstained with DAPI. *Hsp90*, heat shock protein 90; *COX4*, cytochrome c oxidase subunit 4.

The absence of mitochondrial PRDX5 in domestic dog was demonstrated by subcellular fractionation of a liver homogenate. Marker analysis on post-nuclear (E), cytosolic (S), mitochondrial (Mito) and peroxisomal (Perox) fractions was performed by Western blotting (Figure 3A). The results indicated that the cytosolic, mitochondrial and peroxisomal fractions are enriched in Hsp90 (cytosolic marker), COX4 (mitochondrial marker) and catalase (peroxisomal marker) respectively. Evaluation of PRDX5 content by Western blotting revealed the presence of PRDX5 in cytosolic and peroxisomal fractions but PRDX5 was absent from the mitochondrial fraction.

To confirm the absence of mitochondrial PRDX5 in domestic dog, PRDX5 expression was examined using immunofluorescence in the MDCK (dog) cell line. PRDX5 was detected in the cytosol but no colocalization with the mitochondrial marker was observed (Figure 3B–D). PRDX5 detection by immunofluorescence also evidenced the peroxisomal localization of the protein, as illustrated by colocalization with peroxisomal catalase (Figure 3E–G).

Analysis of domestic dog *PRDX5* sequences from GenBank databases (GeneID: 476032) revealed the presence of a single translation initiation codon. This ATG codon corresponds to the second translation initiation site of human *PRDX5* that produces the short form of the protein (S-PRDX5). The first translation initiation codon that enables the synthesis of the long form of PRDX5 (L-PRDX5) was not present in the genomic sequence of domestic dog.

In human, PRDX5 coexists in mitochondria with PRDX3. We confirmed the presence of this PRDX isoform in dog cells by using Western blotting on MDCK cell lysates. Mitochondrial localization of PRDX3 was also verified by immunofluorescence in MDCK cells, as demonstrated by its colocalization with Mitotracker Red (Figure S1).

### L-PRDX5 is Absent in Other Canids

**Figure 4:**
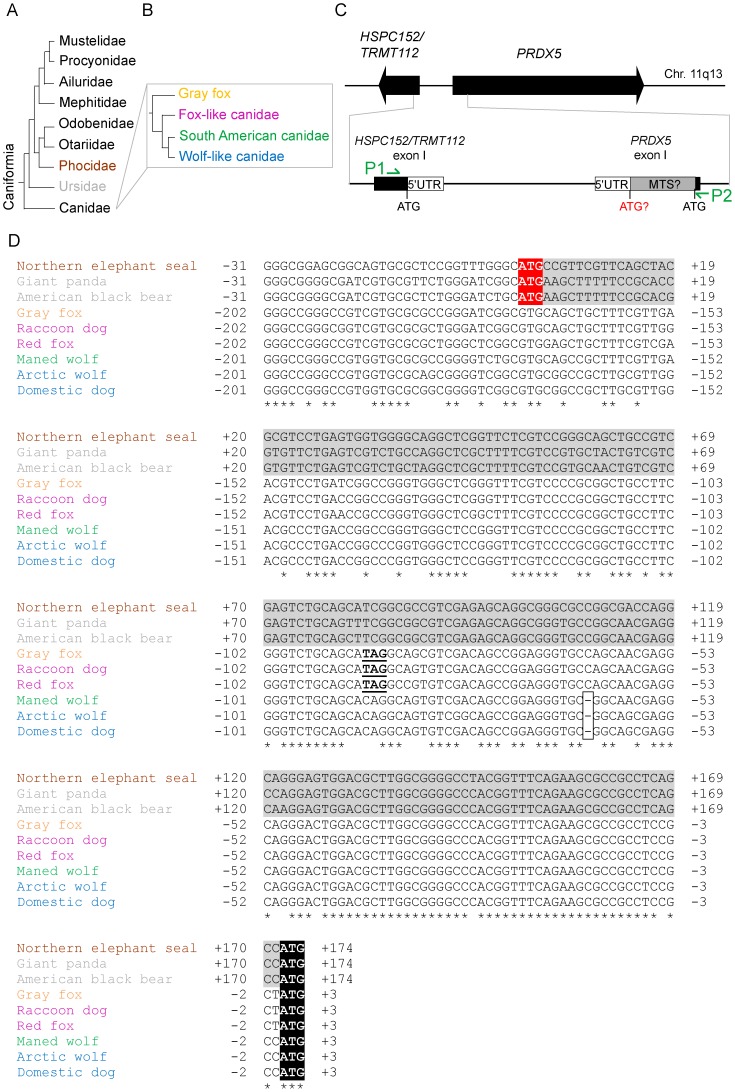
Sequencing and alignment of the *PRDX5* 5′-region in Canidae, Ursidae and Phocidae. (A) Phylogenetic relationships between the nine families composing the suborder Caniformia. The phylogeny of Caniformia is currently not completely established, e.g. concerning the position of Ailuridae. The presented phylogenetic tree is based on multiple nuclear gene sequences and is adapted from [44]. (B) Phylogeny of Canidae, based on ∼15 kb from 12 exons and 4 introns, resolved by [23]. Canidae family contains three major phylogenetic groups, which are indicated in pink for the fox-like canids, green for the South American canids and blue for the wolf-like canids. Additionally, several canids do not belong to any of the three main clades, such as the gray fox (yellow), which was shown to be more primitive. Adapted from [23]. (C) Schematic diagram of *HSPC152/TRMT112* and *PRDX5* genes and cloning strategy. Exons I from both genes are detailed. 5′ UTRs are indicated by open boxes. Gray box highlights the sequence encoding the potential PRDX5 MTS. Conserved translation start sites are indicated in black. The potential translation start site enabling the translation of L-PRDX5 is indicated in red. P1 and P2 indicate the positions to which the PCR primers (green arrows) map. The primers were designated to map on regions sharing 100% identity between giant panda and domestic dog sequences. (D) ClustalX 2.0.7. nucleotidic sequence alignment of *PRDX5* 5′ region from nine Caniformia. Asterisks (*) indicate identities. Sequences encoding a potential MTS are highlighted in gray. Translation start sites enabling the translation of L-PRDX5 and S-PRDX5 are highlighted in red and black respectively. STOP codons being in-frame with the ATG+1 codon are underlined. Gaps are boxed. Species names are written in brown for Phocidae, gray for Ursidae, yellow for gray fox, red for the fox-like canids, green for the South American canids and blue for the wolf-like canids. Only one of the two alleles (Allele 1) found for gray fox and American black bear *PRDX5* are represented. GenBank accession numbers are the following:. Arctic wolf (*Canis lupus arctos*): JQ411232; American black bear (*Ursus americanus)* JQ411225; gray fox (*Urocyon cinereoargenteus*): JQ411227; maned wolf (*Chrysocyon brachyurus*): JQ411231; northern elephant seal (*Mirounga angustirostris*): JQ411224; raccoon dog (*Nyctereutes procyonoides*) JQ411229; red fox (*Vulpes vulpes*): JQ411230. The sequences of domestic dog and giant panda *PRDX5* 5′ region were downloaded from the Genbank references GeneID:476032 (domestic dog, *Canis lupus familiaris*) and GeneID:100473172 (giant panda, *Ailuropoda melanoleuca*).

To date, giant panda (Ursidae) is the phylogenetically closest related species to domestic dog (Canidae) for which *PRDX5* mRNA sequence is available in Genbank databases. These two species belong both to the Caniformia phylum (Figure 4A). Analysis of the 5′ region of giant panda *PRDX5* mRNA (GenBank ID: XM_002916664) revealed the existence of two in-frame translation initiation codons (AUG+1 and AUG+172). These two codons are presumed to lead to the translation of both L-PRDX5 and S-PRDX5. Moreover, subcellular prediction programs predicted a mitochondrial distribution for giant panda L-PRDX5 protein (TargetP: 0.804 and MitoProt II: 0.859 compared with human PRDX5 TargetP: 0.830 and MitoProt II: 0.832; [21], [22]). Since the translation initiation codon of L-PRDX5 appears to be maintained in giant panda but not in domestic dog, the loss of this translation initiation site was likely to have occurred in additional species which are phylogenetically positioned between domestic dog and giant panda, like the other canids (Figure 4A). Canidae family contains three major phylogenetic groups: the fox-like canids, the South American canids and the wolf-like canids (Figure 4B). Additionally, several canids do not belong to any of the three main clades, such as the gray fox, which was shown to be more primitive [23]. To assess the presence of a sequence encoding an MTS in *PRDX5* of other canids, the *PRDX5* 5′ region of gray fox, raccoon dog (fox-like canid), red fox (fox-like canid), maned wolf (South American canid) and arctic wolf (wolf-like canid) was sequenced. Moreover, *PRDX5* 5′ region was also sequenced in northern elephant seal (Phocidae) and American black bear (Ursidae). To obtain the sequence of *PRDX5* 5′ region in these species, the sequence was first PCR-amplified using genomic DNA as template (Figure 4C). The primers were designated to map on regions sharing 100% identity between giant panda and domestic dog sequences. PCR products were cloned in pCR2.1 vector and were subsequently sequenced. Genbank accession numbers are presented in Table 1. The alignment of the 5′ *PRDX5* sequences revealed that the ATG+1 of giant panda, northern elephant seal and American black bear *PRDX5* corresponds to a GTG codon in all the studied canids (Figure 4D). Moreover, in gray fox and fox-like canids, a STOP codon (position−90) is present in the same reading frame as the ATG+1. Furthermore, in the South American and wolf-like canids *PRDX5*, the GTG-171 codon is not in the same reading frame as the ATG+1 codon. In the nucleotidic alignment, this shift in the reading frame corresponds to a gap between nucleotides−64 and−63. It is also worth noting that two slightly different sequences, corresponding to two different alleles, were found in gray fox and American black bear *PRDX5* 5′ region (Table 1).

**Table 1:** Sequences of PRDX5 5′flanking region: Genbank accession numbers.

Species	Accession number
Northern elephant seal	JQ411224
American black bear (Allele 1)	JQ411225
American black bear (Allele 2)	JQ411226
Gray fox (Allele 1)	JQ411227
Gray fox (Allele 2)	JQ411228
Raccoon dog	JQ411229
Red fox	JQ411230
Maned wolf	JQ411231
Arctic wolf	JQ411232

### A functional MTS is Present in Giant Panda and Northern Elephant Seal PRDX5

**Figure 5:**
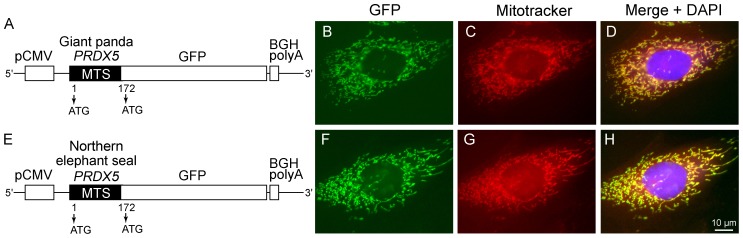
Subcellular localization of giant panda (A–D) and northern elephant seal (E–H) PRDX5 MTS expressed in fusion with GFP in MDCK cells. Structure of the constructs cloned into mammalian expression vector pcDNA3.1 and used for MDCK transfection (A and E). Both alternative ATG initiation codons are indicated (arrows). Transfected cells were examined for GFP fusion expression (B and F). Mitochondria were stained with Mitotracker Red (C and G) and nuclei were counterstained with DAPI. *BGH polyA*, bovine growth hormone polyadenylation signal; *GFP*, green fluorescent protein; *MTS,* mitochondrial targeting sequence; *pCMV*, promoter of cytomegalovirus.

Given that giant panda and northern elephant seal *PRDX5* present two in-frame ATG codons, it was interesting to assess whether the region comprised between these codons encodes a functional MTS. To this end, two pcDNA3.1 plasmids containing the sequence encoding the 58 N-terminal residues of giant panda or northern elephant seal PRDX5 fused with the green fluorescent protein (GFP) were generated (Figure 5A and E). After transient transfection of MDCK cells with these vectors, the reporter protein was detected in mitochondria, as demonstrated by co-localization with Mitotracker Red (Figure 5 B–D and F–H).

### Human L-PRDX5 Makes MDCK Cells More Vulnerable to Acute Oxidative Stress

**Figure 6:**
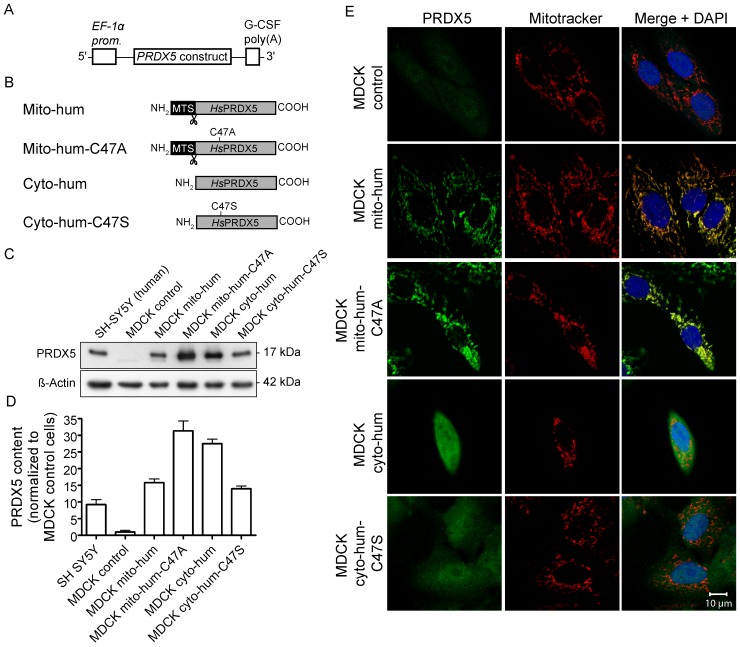
Overexpression of human PRDX5 in MDCK cells. (A) Organization of the construct cloned into mammalian expression vector pEF-BOS. EF-1α prom, promoter region of human EF-1α chromosomal gene; G-CSF poly(A), polyadenylation signal from human granulocyte colony-stimulating factor. (B) Representation of the proteins encoded by the different constructs used for transfection. Mito-hum and Mito-hum-C47A correspond to the enzymatically active and inactive mitochondrial human PRDX5 with the cleavable (scissors) presequence (MTS), respectively. Cyto-hum and Cyto-hum-C47S correspond to enzymatically active and inactive cytosolic human PRDX5, respectively. PRDX5 content of each MDCK clone was verified by Western blotting (C) and total expression levels were quantified (D). PRDX5 protein levels were normalized with β-actin and were expressed in relative units of PRDX5 content in MDCK control cells. Values are means ± SEM of triplicates. The subcellular localization of PRDX5 in the MDCK clones was verified by immunofluorescence (E). Mitochondria were stained with MitoTracker Red prior to cell fixation. Cell nuclei were counterstained with DAPI. *Hs*PRDX5: human PRDX5.

Since PRDX5 is absent in dog mitochondria, it was interesting to assess the functional consequences of the restoration of mitochondrial localization of the protein in MDCK (dog) cells. pEF-BOS was used as mammalian expression vector (Figure 6A). Figure 6B presents the proteins encoded by the constructs used for MDCK transfection. pEF-BOS-mito-PRDX5 and pEF-BOS-cyto-PRDX5 vectors encode human L-PRDX5 (Mito-hum) and S-PRDX5 (Cyto-hum) respectively and were described previously [13]. pEF-BOS-mito-PRDX5-C47A and pEF-BOS-cyto-PRDX5-C47S vectors encode the enzymatically inactive human L-PRDX5 (Mito-hum-C47A) and S-PRDX5 (Cyto-hum-C47S) respectively. The two latter were used in experiments which are described in the following section.

After transfection of MDCK cells with the empty expression vector (control cells), with pEF-BOS-mito-PRDX5 or with pEF-BOS-cyto-PRDX5 vectors, stable clones were selected. The clones were named MDCK control, MDCK mito-hum or MDCK cyto-hum, according to the name of the proteins encoded by the expression vectors. Overexpression of PRDX5 was quantified by immunoblotting (Figure 6C). Immunoblots of total cell lysates revealed a 15- to 30-fold overexpression compared to the MDCK control cells, and a 1.5–to 3-fold higher PRDX5 content compared to human SH-SY5Y cells (Figure 6D). The latter cells contain endogenous mitochondrial PRDX5 [24]. The intracellular localization of PRDX5 was verified by immunofluorescence (Figure 6E). In the MDCK mito-hum cells, PRDX5 was largely located in mitochondria, while MDCK cyto-hum cells strongly expressed PRDX5 in the cytosol. Incorporation of puromycin resistance gene in the genome of MDCK control cells was verified by PCR on genomic DNA (data not shown).

The expression of TXN2, the known mitochondrial physiological reductant of PRDX5, was also assessed to confirm that MDCK mitochondria have the proper system to reduce efficiently human PRDX5. Immunofluorescence detection of TXN2 evidenced that the enzyme is indeed present in MDCK mitochondria (Figure S1). Moreover, comparison of endogenous TXN2 expression levels between MDCK and human SH-SY5Y cells using Western blotting, revealed that expression levels are comparable in the two cell lines (Figure S1).

**Figure 7:**
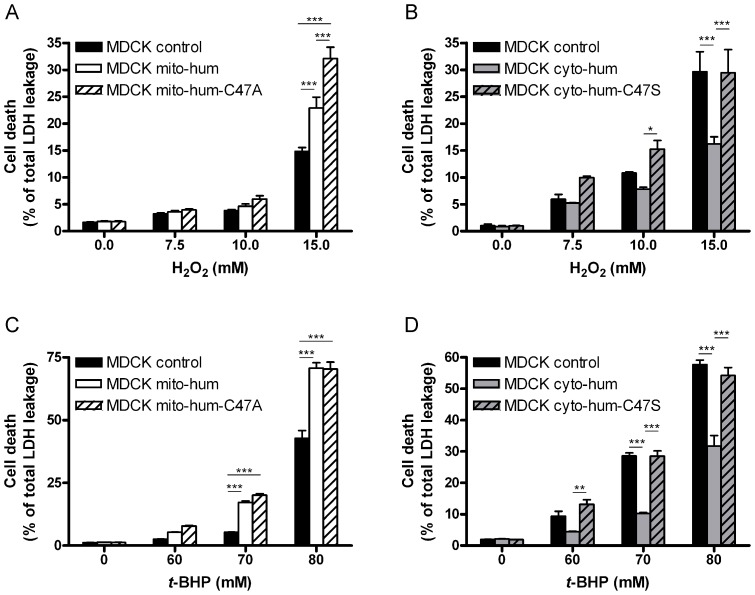
Cytotoxicity induced by hydrogen peroxide (A–B) and *t*-BHP (C–D) in MDCK cells overexpressing human PRDX5. After one hour exposure to high levels of peroxide, cell death was evaluated by LDH release. The total LDH release (100% cell death) was determined after lysis of the cells with 2% Triton X-100. Assays were performed three times for each clone and values are means ± SEM. Significance is designated as *p<0.05, **p<0.01, ***p<0.001. *t-*BHP, *tert*-butyl hydroperoxide.

The MDCK clones were exposed to acute oxidative stress with increasing concentrations of H_2_O_2_ or *t-*BHP (Figure 7). In basal conditions, the MDCK cells expressing human PRDX5 exhibited no significantly different LDH release compared to control cells. Cell death increased with H_2_O_2_ and *t-*BHP exposure. Surprisingly, cell death was significantly more important in MDCK mito-hum cells compared to control cells at 15 mM H_2_O_2_ and 70–80 mM *t-*BHP. On the contrary, cell death was significantly decreased in MDCK cyto-hum cells compared to control cells at high concentrations of H_2_O_2_ and *t-*BHP.

### Increased Cell Death of MDCK Mito-hum Cells During Acute Oxidative Stress is not Linked to PRDX5 Peroxidatic Activity

Since human L-PRDX5 provoked increased cell mortality during acute peroxide-induced oxidative stress in MDCK cells, we assessed whether this deleterious effect was linked to PRDX5 peroxidatic activity. This latter depends on the presence of a peroxidatic cysteine (Cp, see Figure 1). Therefore, MDCK mito-hum-C47A cells were generated, expressing human L-PRDX5 with the Cp replaced by an alanine (C47A mutation; Figure 6B). This mutation inactivates the peroxidatic activity of the enzyme. MDCK cyto-hum-C47S cells were also generated, expressing the enzymatically inactive human S-PRDX5 with the C47S mutation. After transfection and puromycin resistance selection, stable clones were selected and overexpression was quantified by immunoblotting (Figure 6C). Immunoblots of total cell lysates revealed a 10–to 30-fold overexpression compared to MDCK control cells, and a 1.5–to 3-fold higher PRDX5 content compared to human SH-SY5Y cells (Figure 6D). The intracellular localization of PRDX5 was verified in MDCK clones by immunofluorescence (Figure 6E). In the MDCK mito-hum-C47A cells, PRDX5 was largely located in the mitochondria, while MDCK cyto-hum-C47S cells strongly expressed PRDX5 in the cytosol.

MDCK cell lines were exposed to acute oxidative stress induced by H_2_O_2_ or *t-*BHP exposure (Figure 7). Cell death was significantly more important in MDCK mito-hum-C47A cells compared to control cells at 15 mM H_2_O_2_ and 70–80 mM *t-*BHP (Figure 7A and 7C). On the other hand, transient overexpression of a non-specific mitochondrial protein (GFP) in MDCK mitochondria did not make dog cells more sensitive to 7.5–15 mM H_2_O_2_ or 60–80 mM *t*-BHP (Figure S2) and expression of Cyto-hum-C47S protein had no effect on cell viability following H_2_O_2_ or *t-*BHP exposure.

## Discussion

In this work, we show that mitochondrial PRDX5 (L-PRDX5) is absent in domestic dog although cytosolic/peroxisomal PRDX5 (S-PRDX5) is conserved. Sequence analysis of the *PRDX5* 5′ region revealed that the abolition of L-PRDX5 in domestic dog results from the loss of one nucleotide as well as the mutation of the first ATG initiation codon. To determine whether the abolition of mitochondrial PRDX5 occurred only in dog, we analyzed the *PRDX5* 5′ region of several species which are phylogenetically (see Figure 4) closely related to dog and Canidae: giant panda (Ursidae), American black bear (Ursidae) and northern elephant seal (Phocidae). We found that, unlike in domestic dog, the translation initiation codon allowing the synthesis of L-PRDX5 is maintained in these three species. Moreover, we experimentally confirmed that the 58 N-terminal residues of giant panda and northern elephant seal L-PRDX5 are able to target the GFP reporter protein into mitochondria. This indicates that the N-terminal region of giant panda and northern elephant seal L-PRDX5 is a functional MTS. Since PRDX5 MTS is abolished in domestic dog but neither in giant panda nor in northern elephant seal, we investigated the existence of an MTS in PRDX5 in other canids. It appeared that the first ATG codon is mutated in a GTG codon in PRDX5 in all canids that were examined. From these results, we conclude that the loss of mitochondrial PRDX5 is generalized among the extant canids. Moreover, in gray fox, raccoon dog and red fox *PRDX5*, a STOP codon at position−90 is present in the same reading frame as the translation initiation codon of S-PRDX5 (TCG/TAG mutation). Thus, in these more remote canids, two factors impede L-PRDX5 synthesis: the absence of L-PRDX5 translation initiation codon and the presence of a STOP codon. Taken together, sequence analyses suggest that the 5′ region of *PRDX5* in canids corresponds to a sequence that was able to encode a presequence ancestrally. The first events leading to PRDX5 MTS abolition in canids would have involved the mutation of the first translation initiation codon (ATG/GTG mutation) as well as the apparition of a STOP codon (TCG/TAG mutation). However, from our sequence analyses, we cannot conclude which of these two events occurred first during evolution. Furthermore, in South American canids (maned wolf) and wolf-like canids (domestic dog and arctic wolf), the ATG/GTG mutation would have been maintained but the STOP codon would further have evolved to a CAG codon. Also, in the ancestral PRDX5 MTS from these species, a single nucleotide would have been lost. Finally, our sequence analyses indicate that the abolition of PRDX5 MTS would have occurred after the divergence of Canidae from the other Caniformia members. The Canidae family diverged from other Caniformia about 50 million years ago (mya), but extant canids have diverged from a common ancestor within the last 10 million years [25]. Therefore, the loss of mitochondrial PRDX5 in canids would have occurred between 50 and 10 mya.

In a recent report, we have shown that domestic pig has also lost mitochondrial PRDX5 according to a similar molecular mechanism, namely by the loss of the translation initiation codon of L-PRDX5 [19]. Since mitochondrial PRDX5 is maintained in bovine, we concluded that PRDX5 MTS was lost during Cetartiodactyla evolution. Therefore, the loss of L-PRDX5 in canids and domestic pig results from two distinct events during mammalian evolution, i.e. one having occurred during Caniformia diversification, the other taking place during Cetartiodactyla evolution. Given that PRDX5 distribution was analyzed in a limited number of species so far, we cannot exclude that its subcellular distribution would have changed at more occasions during the evolution of mammals. Accordingly, it would be interesting to study PRDX5 subcellular distribution in additional phylogenetic groups. To our knowledge, few enzymes have been reported to have changed their subcellular distribution during mammalian evolution. One example concerns the intermediary metabolic enzyme alanine:glyoxylate aminotransferase (AGT), whose subcellular localization was reported to change during mammalian evolution [26], [27]. Interestingly, the molecular mechanism responsible for the loss of mitochondrial targeting in AGT and PRDX5 is very similar.

The abolition of PRDX5 MTS in canids shows that mitochondrial PRDX5 is not essential in canid species. Some of these species have encountered high evolutionary success, like the raccoon dog or the red fox. It appears thus that the absence of mitochondrial PRDX5 does not impair species’ ability to meet a huge evolutionary success. The abolition of mitochondrial PRDX5 in canids is nevertheless surprising. Indeed, an MTS is conserved in various other vertebrate and invertebrate species [2], [12]–[19], even belonging to basal metazoan phyla, like placozoa and porifera (see Figure 2). Moreover, the cytoprotective function of mitochondrial PRDX5 was described in several studies. For example, it has been shown that overexpression of L-PRDX5 confers resistance to peroxide or drug-induced DNA damage and cell death [13], [28]–[31]. The abolition of L-PRDX5 is unexpected since mitochondria are particularly exposed to oxidative attacks caused by ROS and RNS [1] but we also confirm here that PRDX3 is conserved in dog mitochondria (Supporting Information). Since mitochondrial PRDX5 is absent in dog, it was interesting to investigate the functional consequences of the restoration of L-PRDX5 in dog cells. In order to evaluate the ability of mitochondrial PRDX5 to afford antioxidant protection in dog during oxidative stress, we generated MDCK (dog) cell lines expressing human mitochondrial PRDX5. Moreover, to compare PRDX5 ability to exert its cytoprotective effect according to subcellular localization, we also generated MDCK expressing human cytosolic/peroxisomal PRDX5. Surprisingly, PRDX5 expression in MDCK mitochondria resulted in an increased cell mortality in cells exposed to high peroxide concentrations, while PRDX5 expression in MDCK cytosol/peroxisomes afforded a greater resistance to acute oxidative stress. As mentioned before, L-PRDX5 differs from S-PRDX5 by the presence of an additional N-terminal MTS. However, this MTS is cleaved after mitochondrial import, releasing a functionally active 17 kDa mature mitochondrial PRDX5 which is identical to S-PRDX5 [6]. In MDCK mito-hum cells, no PRDX5 signal at higher molecular weight was detected by Western blotting, indicating that the MTS was removed efficiently. Therefore, it appears that mitochondrial and cytosolic/peroxisomal human PRDX5, although being identical, do not equally protect MDCK cells, depending on their subcellular localization. Moreover, PRDX5 does not only appear to be an ineffective antioxidant protective enzyme in MDCK mitochondria–which would have resulted in an unchanged cell viability during oxidative stress–but this protein seems also to gain cytotoxic properties under acute oxidative stress, resulting in an increased cell mortality. Furthermore, given that MDCK mito-hum cells exhibit an increased cell death during oxidative stress but not under basal conditions, one would have hypothesized that the sensitization was linked to PRDX5 peroxidatic activity. However, MDCK cells expressing enzymatically inactive mitochondrial PRDX5 exhibited also a higher cell mortality following oxidative stress, indicating that human PRDX5 provokes deleterious effects in MDCK mitochondria independently of its enzymatic activity. Thus, it appears that the protein, rather than its peroxidatic activity, is inappropriate in the MDCK mitochondrial matrix during acute oxidative stress. At this stage, it is difficult to establish which molecular mechanism is responsible for the peroxide-induced sensitization of the MDCK mito-hum cells. We could hypothesize that PRDX5 cytotoxic property is linked to a redox-dependent interaction with another MDCK mitochondrial protein, impairing the function of the binding partner. Interactions of PRDXs with various proteins have been described. Such interactions have been shown to modulate the functions of the binding partners [32]. However, so far, little is known about PRDX5 interactions with mitochondrial proteins. Moreover, since expression of human PRDX5 in MDCK mitochondria led to high overexpression (Fig. 6C), a non-specific protein effect could have been considered but overexpression of GFP in MDCK mitochondria did not increase cell sensitivity to H_2_O_2_ and *t*-BHP (Figure S2). It must be also noted that, in a previous study, we used the same expression vectors to assess the cytoprotective function of human L-PRDX5 and S-PRDX5 in Chinese hamster ovary (CHO) cells [13]. In these cells, which contain endogenous mitochondrial PRDX5, both L-PRDX5 and S-PRDX5 afforded antioxidant protection during acute oxidative stress. It appears thus that human L-PRDX5 triggers deleterious effects during acute oxidative stress in cells lacking endogenous mitochondrial PRDX5, while the same protein confers antioxidant resistance in cells possessing endogenous mitochondrial PRDX5. This led us to suggest that, despite the fact that PRDX5 cytoprotective function has been clearly demonstrated by several studies in human and rodents, the conservation of mitochondrial PRDX5 would not confer necessarily an advantage in all mammalian species. Also, although the loss of L-PRDX5 in canids could have occurred independently of this phenomenon, it is tempting to speculate that the cytotoxic property of L-PRDX5 in mitochondria is at the origin of the abolition of mitochondrial PRDX5 in canids.

Interestingly, analysis of PRDX5 sequences from different animal species reveals that the non-conservation of PRDX5 mitochondrial targeting represents only a fraction of PRDX5 subcellular variability that is found through animal kingdom (see Figure 2). Indeed, in addition to the lack of PRDX5 MTS in some mammals, the PTS1, responsible for peroxisomal localization, appears also to be abolished in some animal species. Moreover, the presence of the second translation initiation codon, permitting the translation of S-PRDX5, appears to be absent in some species, like in bay scallop. In these species, only L-PRDX5 is likely to be translated. Taken together, PRDX5 sequence analyses suggest that PRDX5 subcellular relocalizations occurred at many occasions during animal evolution. Whether this variability permits an adaptation to specific needs, metabolic pathways or life environments remains a matter of speculation.

To conclude, the present study shows that mitochondrial PRDX5 has been lost during canid evolution. Our results suggest that the abolition of mitochondrial PRDX5 in the Canidae family would have occurred between 50 and 10 mya and involves the mutation of the first translation initiation codon of PRDX5 as well as the appearance of a STOP codon in the ancestral MTS. Finally, our work shows also that the restoration of mitochondrial PRDX5 in MDCK cells, even devoid of its peroxidatic activity, triggers deleterious effects during acute oxidative stress conditions.

## Materials and Methods

### Computer Analysis

Multiple sequence alignment was performed with ClustalX 2.0.7 [33]. TargetP 1.1 and MitoProt II v.1.101 were used for MTS prediction [21], [22].

### Dog Liver Fractionation

Fresh liver was collected from beagle dog under the guidelines of the European Convention on the Protection of Vertebrate Animals [34]. Dog liver was homogenized and centrifuged to obtain nuclear fractions (N) and postnuclear supernatants (E). E-fraction was further fractionated by differential centrifugation into heavy mitochondrial (M), light mitochondrial (L), microsomal (P) and cytosolic (S) fractions. Subfractions of the L-fraction were obtained by centrifugation through a Percoll gradient. Marker enzymes and protein content were measured in each fraction. Based on glutamate dehydrogenase (GDH, mitochondrial marker) and catalase (peroxisomal marker) activities, Percoll fractions 2 and 10 were chosen to represent mitochondrial (Mito) and peroxisomal (Perox) fractions respectively. Experimental procedures have been described previously [35], [36].

### Western Blotting

Western blotting was performed as described in [19]. Blots were probed overnight with 1∶4000 polyclonal rabbit anti-human PRDX5 [37], 1 µg/ml monoclonal mouse anti-heat shock protein 90 (Hsp90; Millipore), 1∶2000 monoclonal mouse anti-cytochrome c oxidase subunit 4 (COX4; Molecular Probes), 1∶3000 polyclonal rabbit anti-catalase (Rockland) or 1∶1500 polyclonal rabbit anti-actin (Sigma). Subsequently, blots were incubated for one hour with peroxidase-conjugated goat anti-rabbit IgG or goat anti-mouse IgG (Dako). Blots were developed with the Western Lightning Chemiluminescence kit (PerkinElmer) according to manufacturer’s instructions.

### Cell Culture

MDCK (dog kidney) cell line was cultured in Dulbecco’s Modified Eagle medium (DMEM) containing 4.5 g/l D-glucose with GlutaMAX (Gibco), supplemented with 10% foetal calf serum (FCS; Gibco), 1% non-essential amino acids, 100 U/ml streptomycin and 100 U/ml penicillin (Gibco). Cells were maintained at 37°C in 5% CO_2_.

### Immunofluorescence

Mitotracker (Molecular Probes) staining and immunofluorescence were performed as described previously [19], using 1∶200 rabbit anti-human PRDX5 and 1∶50 FITC-conjugated donkey anti-rabbit IgG (Jackson ImmunoResearch Laboratories Inc.). Co-localization experiment with catalase was performed using 1∶200 rabbit anti-human PRDX5, 1∶100 sheep anti-human catalase (SanBio), 1∶50 TRITC-conjugated donkey anti-rabbit IgG (Jackson ImmunoResearch Laboratories Inc.) and 1∶50 FITC-conjugated donkey anti-sheep IgG (Jackson ImmunoResearch Laboratories Inc.). The coverslips were mounted in Vectashield (Vector Laboratories, Inc.) containing 50 µg/ml 4,6-diamidino-2-phenylindole dihydrochloride (DAPI) and analyzed by fluorescence microscopy.

### Sequencing the 5′ Region of *PRDX5* from Caniformia Species

The species examined in this experiment are the following: arctic wolf (*Canis lupus arctos*), American black bear (*Ursus americanus*), domestic dog (*Canis lupus familiaris*), giant panda (*Ailuropoda melanoleuca*), gray fox (*Urocyon cinereoargenteus*), maned wolf (*Chrysocyon brachyurus*), northern elephant seal (*Mirounga angustirostris*), raccoon dog (*Nyctereutes procyonides*) and red fox (*Vulpes vulpes*). Arctic wolf blood sample was kindly provided during a routine blood checkup by Dr. JC Bertho, zoological park “Le Monde Sauvage”, Aywaille, Belgium. American black bear heart sample has been used in [38] and was generously given by Pr. MAG Van Der Heyden (University Medical Center Utrecht, The Netherlands). Gray fox and maned wolf DNA samples, described in [23], were kindly provided by Pr. RK Wayne (University of California, USA). Northern elephant seal blood sample has been used in [39] and was provided by C Louis and Pr. C Debier (Université catholique de Louvain, Belgium). Tissue samples of raccoon dog and red fox were taken at necropsy of animals that have been found dead accidentally respectively in Finland and in Belgium. No animal was killed for the purpose of this study.

Genomic DNA was extracted from blood or muscle using the DNeasy Blood and tissue Kit (Qiagen) following manufacturer’s instructions. Primers 5′-CCAGCGGTCACCTTGATTGGGGC-3′ (forward) and 5′-ATGCGAGCTCAGCAGGTTGTGG-3′ (reverse) were used to amplify the genomic region comprised between the first exon of *PRDX5* and the first exon of *HSPC152/TRMT112*, which is in close proximity of *PRDX5* and positioned in opposite direction (Fig. 1 and [11]). The primers were designated to map on regions sharing 100% identity between giant panda and dog sequences. The PCR reaction was performed using the Taq DNA polymerase (Eurogentec) in the presence of 5% (v/v) dimethyl sulfoxide (DMSO). The PCR products were cloned into pCR2.1 vector and sequenced. At least eight clones, deriving from three independent PCR reactions, were sequenced for each species. The sequences of the 5′ region of *PRDX5* from caniformia species have been submitted to GenBank (see Table 1).

### Expression of Giant Panda and Northern Elephant seal PRDX5 MTS in Fusion with Green Fluorescent Protein (GFP) in MDCK Cells

Based on Genbank database sequence XM_002916664, the sequence encoding giant panda PRDX5 MTS was synthesized and cloned into pEX-A vector by Eurofins MWG Operon. The sequence coding for giant panda PRDX5 MTS was subsequently PCR-amplified using forward primer 5′-GGGATCGGCATGAAGCTTTTTCCGCACCG-3′ and reverse primer 5′-CCATGGCTGAGGCGGCGCTTCTGAAACC-3′. The sequence encoding the MTS of northern elephant seal PRDX5 was PCR-amplified from a pCR2.1 vector containing northern elephant seal *PRDX5* 5′ flanking region (see point 4.6.) using forward primer 5′-GGTTTGGGCATGCCGTTCGTTCAGCTACG-3′ and reverse primer 5′-CCATGGCTGAGGCGGCGCTTCTGAAACC-3′. PCR products were cloned in the pcDNA3.1/CT-GFP-TOPO vector (Invitrogen) and the resulting plasmids were sequenced. MDCK cells were transiently transfected using Amaxa Nucleofection system (Lonza AG) according to manufacturer’s instructions. 72 h after transfection, cells were incubated with Mitotracker Red prior to fixation. The coverslips were mounted in Vectashield containing 50 µg/ml DAPI and analyzed by fluorescence microscopy.

### Generation of Stable MDCK Clones Expressing Mitochondrial or Cytosolic/Peroxisomal Human PRDX5

Plasmid pEF-BOS containing an EF-1-α promoter, a puromycin resistance gene and G-CSF-poly(A) adenylation signal was used as mammalian expression vector. pEF-BOS-Mito-PRDX5 (*PRDX5* cDNA sequence encoding human L-PRDX5) and pEF-BOS-Cyto-PRDX5 (*PRDX5* cDNA sequence encoding human S-PRDX5) were described previously [13].

For the construction of pEF-BOS-Mito-PRDX5-C47A (*PRDX5* cDNA sequence encoding human L-PRDX5 with the residue Cys47 mutated into Ala) vector, human *PRDX5* cDNA was PCR-amplified from pEF-BOS-Mito-PRDX5 using forward primer 5′-ACAGGGATTTCTTGTCTCCCACG-3′ and reverse primer 5′-TCTCAAGCCTCAGACAGTGG-3′. The C47A mutation was generated by PCR-mediated site-directed mutagenesis using the complementary primers containing a base mismatch 5′- ACCCCTGGAGCTAGCAAGACACACCTG-3′ (forward) and 5′-TGTGTCTTGCTAGCTCCAGGGGTGAAGG-3′ (reverse). The resulting amplicon was ligated in the pCR2.1 vector and subcloned into the *Spe*I and *Not*I sites of pEF-BOS vector.

For the construction of pEF-BOS-Cyto-PRDX5-C47S (*PRDX5* cDNA sequence encoding human S-PRDX5 with the residue Cys47 mutated into Ser) vector, human *PRDX5* cDNA containing the mutation C47S [40] was PCR-amplified using forward primer 5′- AGCGACTAGTCCGCCATGGCCCCAATCAAGGTGGGAGATGC-3′ (*Spe*I site underlined) and reverse primer 5′- TATCTGGCGGCCGCCACTCAGAGCTGTGAGATGATATTGGG- 3′ (*Not*I site underlined). The resulting amplicon was ligated in the pCR2.1 vector and subcloned into the *Spe*I and *Not*I sites of pEF-BOS vector.

MDCK cells were transfected with plasmids using Amaxa Nucleofection system according to manufacturer’s instructions. Two days after transfection, cells were selected 16 days with 2 µg/ml puromycin (Sigma-Aldrich). Subsequently, stably transfected cell lines were obtained by isolation of individual clones by a dilution technique. At least three clones per cell line were obtained and characterized.

Overexpression of PRDX5 was confirmed by immunofluorescence and immunoblotting (Figure 6). Incorporation of puromycin resistance gene in the genome of MDCK control cells was verified by PCR on genomic DNA. The latter was extracted from 5×10^6^ cells using the DNeasy Blood and Tissue kit (Qiagen) following manufacturer’s instructions. The puromycin N-acetyltransferase gene was amplified by using forward primer 5′-GACCGAGTACAAGCCCACGGTGCG-3′ and reverse primer 5′-CGAGACGCC GACGGTGGCCAGGAACCACG-3′.

### Cell Treatment with Hydrogen Peroxide and *Tert*-butyl Hydroperoxide (t-BHP) and Lactate Dehydrogenase (LDH) Assay

MDCK cells were seeded at a density of 3×10^4^ cells/cm^2^ into 24-well plates. After 15–18 h culture, stress was induced during one hour in DMEM without phenol red containing 4.5 g/l D-glucose and supplemented with 1% FBS, 100 U/ml penicillin, 100 U/ml streptomycin, 1% non-essential amino acids and 2 mM GlutaMAX. Cytotoxicity was evaluated by the lactate dehydrogenase (LDH) release assay (Cytotoxicity detection Kit, Roche). Fifty µl of supernatants were used for LDH assay. The total LDH release (100% cell death) was determined for each well after lysis of cells with 2% Triton X-100 detergent. Cell mortality was calculated according to this scale.

### Statistical Analysis

Cell death values were calculated from triplicates within an experiment. Each experiment was repeated three times for one clone, allowing the calculation of means and SEM. Statistical analysis was performed using two-way ANOVA with the Bonferroni post hoc test. Significance is designated as *p<0.05, **p<0.01, ***p<0.001. At least three clones were tested for each cell line.

## Supporting Information

Figure S1 (A) Subcellular localization of PRDX3 and TXN2 was assessed by immunofluorescence detection of endogenous proteins in dog MDCK cells. Mitotracker staining and immunofluorescence were performed as described in section 4.5, using 1∶200 rabbit anti-human PRDX3 [24] and 1∶200 rabbit anti-human TXN2 [41] polyclonal antibodies. Nuclei were counterstained with DAPI. Expression levels of PRDX3 (B) and TXN2 (C) were quantitated and compared in MDCK and SH-SY5Y cells. PRDX3 and TXN2 were detected by Western blotting in soluble proteins from whole cell extracts. Indicated amounts of human recombinant 6xHis-tagged PRDX3 [24] and TXN2 [42] were also blotted for quantitation. Western blotting was performed as described under section 4.3. Blots were probed with 1∶4000 rabbit anti-human PRDX3 and 1∶4000 rabbit anti-human TXN2. PRDX3 (D) and TXN2 (E) expression levels were quantified by comparison with recombinant protein standards. PRDX3 and TXN2 expression levels were expressed as the percentage of total protein content. Values are means ± SEM of triplicates.
(TIF)

Figure S2 Overexpression of a non-specific mitochondrial protein (GFP) in MDCK mitochondria does not make cells more vulnerable to H_2_O_2_ or t-BHP. (A) Representative population of MDCK cells transiently transfected with construct containing giant panda PRDX5 MTS fused to GFP (MDCK mito-GFP) 48 h post-transfection (nuclei stained with DAPI). Scale bar−20 µm. (B) Percentage of GFP positive cells 48 h post-transfection. Count was performed on six randomly chosen fields by slide. Value is the mean ± SEM of two independent slide counts. (C–D) 96 hours post-transfection, cell death was determined by LDH assay following 1 hour exposure to indicated concentrations of H_2_O_2_ (C) or *t*-BHP (D). Total released LDH activity was determined after cell lysis in 2% Triton X-100. Values are means ± SEM from triplicates. Significance versus control (MDCK control cells transfected with empty vector) is indicated by *p<0.05 (two-way ANOVA followed by Bonferroni post-hoc test).
(TIF)
