# Supplementary figures and images for: Abolition of Peroxiredoxin-5 Mitochondrial Targeting during Canid Evolution
Source: PLoS One. 2013 Sep 2;8(9):e72844. doi: 10.1371/journal.pone.0072844 (PMC3759418; doi:10.1371/journal.pone.0072844)

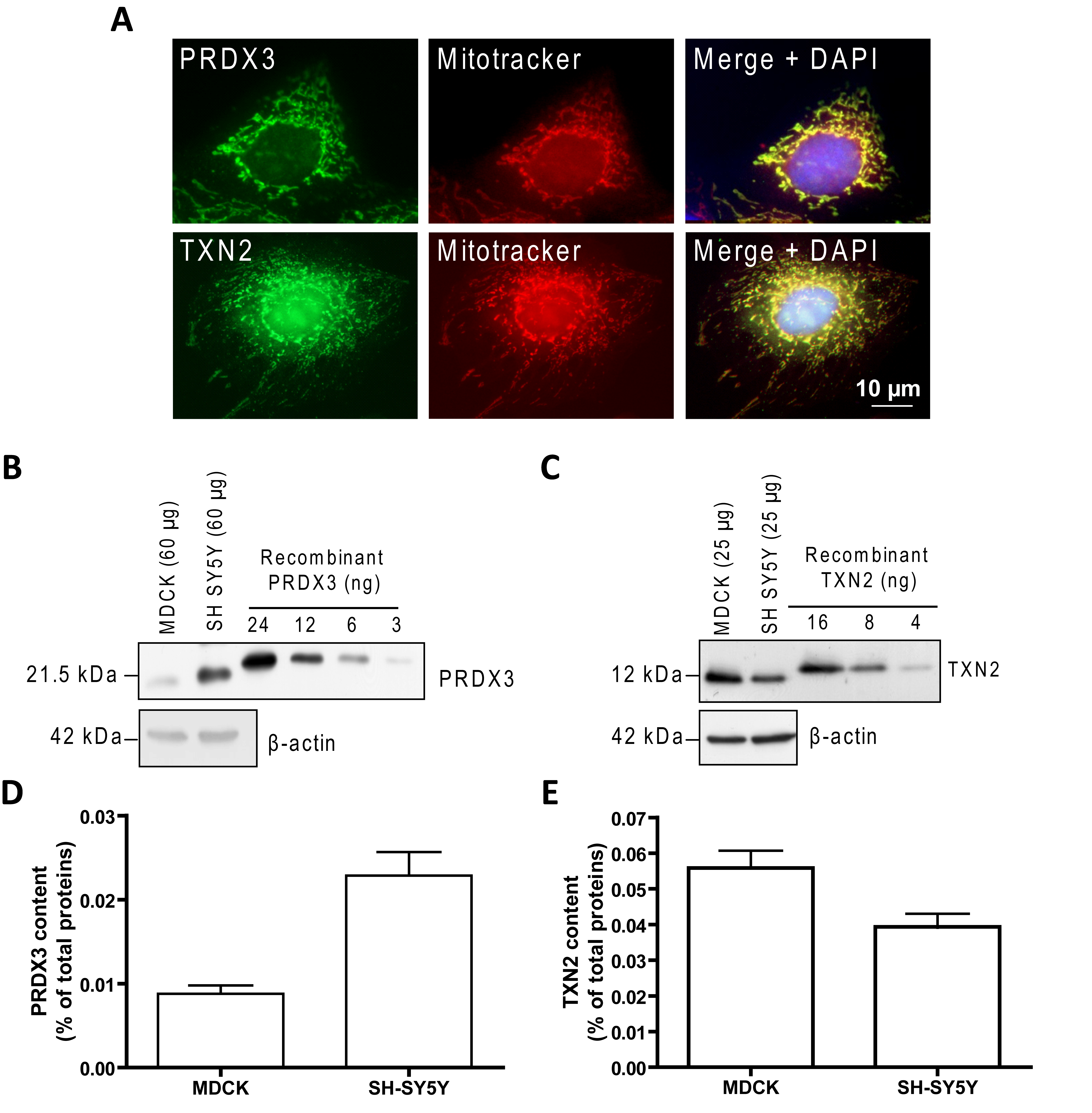

Supplement: Figure S1 — (A) Subcellular localization of PRDX3 and TXN2 was assessed by immunofluorescence detection of endogenous proteins in dog MDCK cells. Mitotracker staining and immunofluorescence were performed as described in section 4.5, using 1∶200 rabbit anti-human PRDX3 [24] and 1∶200 rabbit anti-human TXN2 [41] polyclonal antibodies. Nuclei were counterstained with DAPI. Expression levels of PRDX3 (B) and TXN2 (C) were quantitated and compared in MDCK and SH-SY5Y cells. PRDX3 and TXN2 were detected by Western blotting in soluble proteins from whole cell extracts. Indicated amounts of human recombinant 6xHis-tagged PRDX3 [24] and TXN2 [42] were also blotted for quantitation. Western blotting was performed as described under section 4.3. Blots were probed with 1∶4000 rabbit anti-human PRDX3 and 1∶4000 rabbit anti-human TXN2. PRDX3 (D) and TXN2 (E) expression levels were quantified by comparison with recombinant protein standards. PRDX3 and TXN2 expression levels were expressed as the percentage of total protein content. Values are means ± SEM of triplicates. (TIF) [file pone.0072844.s001.tif]

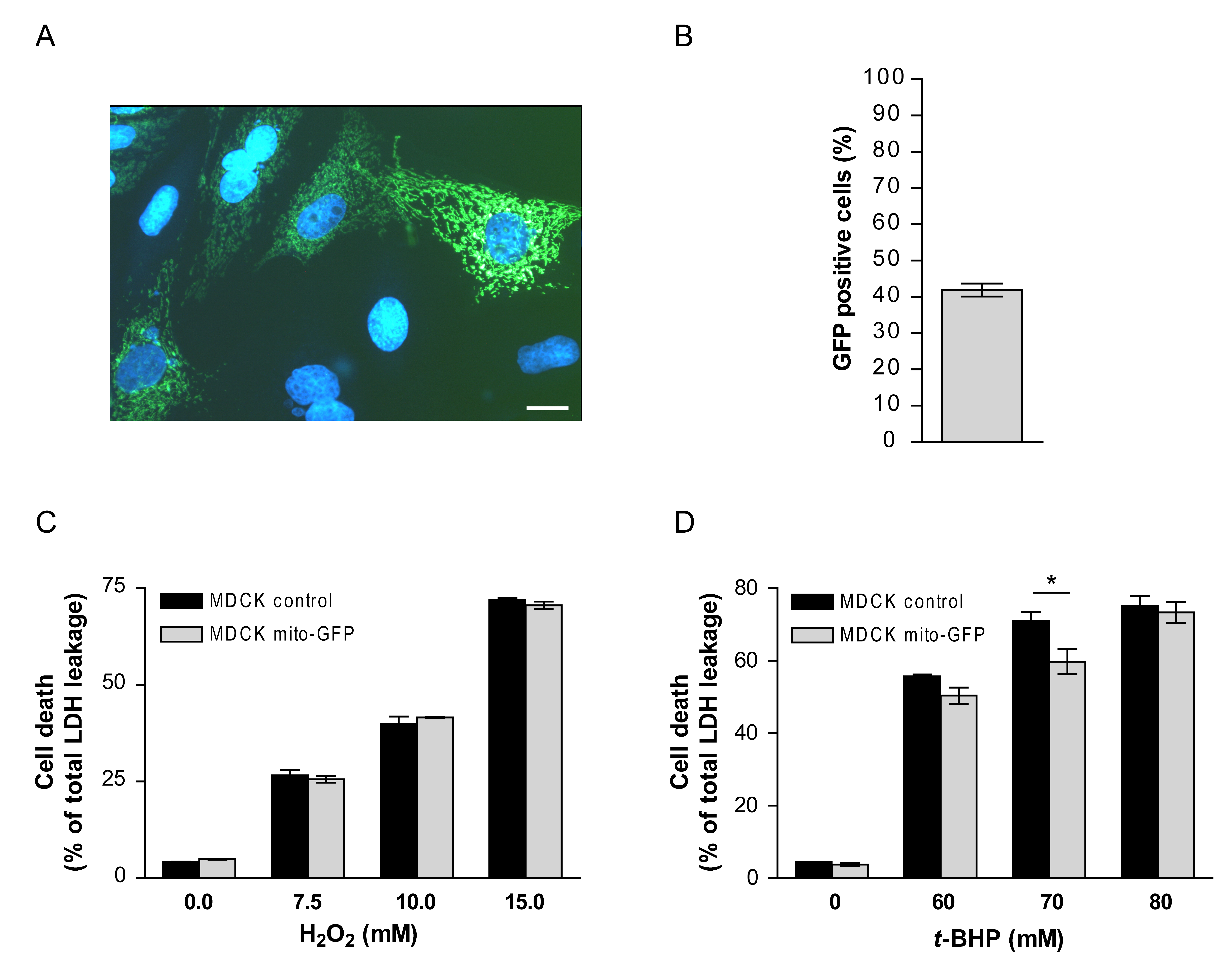

Supplement: Figure S2 — Overexpression of a non-specific mitochondrial protein (GFP) in MDCK mitochondria does not make cells more vulnerable to H2O2 or t-BHP. (A) Representative population of MDCK cells transiently transfected with construct containing giant panda PRDX5 MTS fused to GFP (MDCK mito-GFP) 48 h post-transfection (nuclei stained with DAPI). Scale bar−20 µm. (B) Percentage of GFP positive cells 48 h post-transfection. Count was performed on six randomly chosen fields by slide. Value is the mean ± SEM of two independent slide counts. (C–D) 96 hours post-transfection, cell death was determined by LDH assay following 1 hour exposure to indicated concentrations of H2O2 (C) or t-BHP (D). Total released LDH activity was determined after cell lysis in 2% Triton X-100. Values are means ± SEM from triplicates. Significance versus control (MDCK control cells transfected with empty vector) is indicated by *p<0.05 (two-way ANOVA followed by Bonferroni post-hoc test). (TIF) [file pone.0072844.s002.tif]
